# Antimicrobial resistance trend of bacterial uropathogens at the university of Gondar comprehensive specialized hospital, northwest Ethiopia: A 10 years retrospective study

**DOI:** 10.1371/journal.pone.0266878

**Published:** 2022-04-11

**Authors:** Desie Kasew, Blen Desalegn, Mihret Aynalem, Sosina Tila, Dureti Diriba, Beimnet Afework, Michael Getie, Sirak Biset, Habtamu Wondifraw Baynes

**Affiliations:** 1 Department of Medical Microbiology, School of Biomedical and Laboratory Science, College of Medicine and Health Sciences, University of Gondar, Gondar, Ethiopia; 2 Department of Medical Laboratory Science, School of Biomedical and Laboratory Science, College of Medicine and Health Sciences University of Gondar, Gondar, Ethiopia; 3 Microbiology Laboratory, University of Gondar Comprehensive Specialized Hospital, Gondar, Ethiopia; 4 Department of Clinical Chemistry, School of Biomedical and Laboratory Science, College of Medicine and Health Sciences, University of Gondar, Gondar, Ethiopia; University of Lincoln, UNITED KINGDOM

## Abstract

Urinary tract infection and antimicrobial resistance remains the major problem, with significant health and socioeconomic burden, particularly in developing countries. This infection is commonly caused by Gram-negative bacteria, principally by *Escherichia coli*. So, this study aimed to determine bacterial isolates and antimicrobial resistance trend among patients with urinary tract infection at the University of Gondar Comprehensive Specialized Hospital, Northwest Ethiopia. A retrospective study was conducted from January 1^st^ to February 28^th^. A ten years (2010–2019) record of urine culture results, the biochemical test and antimicrobial susceptibility test results of isolates were collected from the medical microbiology laboratory register using a checklist. Data quality was checked, entered, and analyzed using SPSS version 23. We have presented results through descriptive tables and graphs. The overall prevalence of urinary tract infection among 4441 patients was 24.1%. *Escherichia coli* (37.7%), *Klebsiella pneumoniae* (11.4%), and *Staphylococcus aureus* (9.1%) were the predominant uropathogens. The infection rate was nearly similar across both sexes but highest in the age group above 60 years. Above 75% of Gram-negative isolates were resistant to ampicillin (92.5%), amoxicillin-clavulanate (80.1%), tetracycline (79.3%), cefuroxime (79.2%), and Trimethoprim-sulfamethoxazole (78.3%). Over 2/3 of Gram-positive isolates also showed increased resistance to tetracycline (84.8%) and penicillin (71.6%). Moreover, more than 44% of the isolates were multidrug-resistant (MDR). We have seen an inconsistent trend of antimicrobial resistance, with an overall resistance rate of above 50%. In conclusion, the overall prevalence of urinary tract infection was high and elderly patients were most affected. More than 70% of both Gram positive and gram-negative isolates were resistant to penicillin, ampicillin, amoxicillin-clavulanate, tetracycline, cefuroxime, Trimethoprim-sulfamethoxazole. Above than 44% of the isolates were multidrug-resistant (MDR). The increasing rate of antimicrobial resistance calls for routine diagnosis and antimicrobial susceptibility testing. A prospective multicenter study indicating the status of resistance should be encouraged.

## Introduction

Urinary tract infection (UTI) is one of the most common infectious diseases, particularly in developing countries, overwhelmed with healthcare and economic constraints [1]. Urinary tract infection can be called pyelonephritis (kidney infection), or cystitis (bladder infection). The infection has clinical signs and symptoms such as dysuria, frequency, urgency, suprapubic tenderness, fever, chills, nausea, and vomiting [2, 3]. The bacterial causes of UTI include *Escherichia coli (E*. *coli)* (which causes 80% of the UTI), *Klebsiella pneumoniae (K*. *pneumoniae)*, *Citrobacter* species, *Enterobacter* species, *Pseudomonas aeruginosa (P*. *aeruginosa)*, and *Staphylococcus* species [4–6]. The mechanism of pathogenesis of the mentioned pathogens include adhesion to the host cell epithelium, invasion, immune evasion via cell wall lipopolysaccharide, capsule, and fimbriae [7]. The infection is higher in females due to biological factors such as the short urethra, anal-genital proximity, and use of spermicides [8].

Urinary tract infection is associated with increased resistance to antimicrobial agents such as multidrug resistance (MDR) with substantial medical and a financial burden [9, 10]. Antimicrobial resistance is a thoughtful medical problem in which microorganisms use varied resistance mechanisms such as horizontal gene transfer (such as plasmids and bacteriophages), genetic recombination, and mutations [11]. In addition, self-medication [12], empirical therapy, misuse, and overuse of antimicrobials which are highly practiced in Ethiopia, hasten antimicrobial resistance (AMR) end up in prolonged illness, disability, increased health care costs, and death [1, 13, 14]. In the era of rising antimicrobial resistance, current longitudinal studies revealing the prevalence and AMR trend of uropathogens are crucial to coming up with this problem [15]. This up-to-date evidence will support clinicians to identify the etiology of UTI, ensure appropriate empirical treatment for a reasonable period and an affordable cost. Moreover, it helps health policymakers in implementing locally efficient therapy and preventive guidelines. Although there are snapshot studies on the prevalence of UTI and associated AMR, data showing results of longitudinal studies lacked in the study area. Hence, this study aimed to assess the prevalence and AMR trend of bacterial uropathogens over 10 years between 2010 and 2019 among patients with UTI at the University of Gondar Comprehensive Specialized Hospital (UoGCSH), Northwest Ethiopia.

## Materials and methods

### Study area, design and period

A Hospital-based retrospective study was conducted by retrieving laboratory record of ten years (2010–2019) data from January 1^st^ to February 28^th^, 2020 at the UoGCSH, Gondar, Ethiopia. The University hospital is one of the pioneering tertiary level referral and teaching hospitals in the country, which serves more than 5 million people in Gondar province and neighboring regions. It has different service centers in inpatient and outpatient settings such as fistula, cancer, dialysis, psychiatric and ophthalmology clinics. It also has an organized laboratory such as microbiology and mycobacteriology sections [16]. We have collected manually, a ten years (2010–2019) retrospective data from the microbiology laboratory logbook which is a paper-based record of the laboratory results. We have collected data complete record of the variables mentioned in the exclusion criteria.

All recorded urine culture results of patients who visited the UoGCSH and were suspected of UTI were the source. Moreover, the recorded urine culture results of those UTI suspected patients who visited the hospital from 2010–2019 were the study population and analyzed.

Inclusion and exclusion criteria: We have included the records of patients’ data which contains the patients’ age, sex, urine culture result including antimicrobial susceptibility test (AST) results for significant bacteriuria (10^5^ CFU/ml) of monomorphic organisms which have been processed and recorded from 2010 to 2019. However, records lacking at least one of the variables age, sex, urine culture results, and AST results of cultures with significant bacteriuria were excluded.

Ethical approval letter was obtained from ethical review committee (ERC) of school of Biomedical and Laboratory Sciences, College of medicine and health sciences, University of Gondar. We explained the study objectives to the heads of the hospital director and laboratory personnel who worked in the hospital. Consent from patients was not obtained as a waiver of consent by the ERC. In addition, we extracted our research data from a record in which patients’ name was anonymous.

### Data collection and analysis

We have collected variables such as age, sex, urine culture result, isolated uropathogens, and their AST results from the UoGCSH Microbiology laboratory record book by using a data collection checklist. The urine specimen was first collected with sterile wide mouthed cup and inoculated on Cysteine-Lactose-Electrolyte-Deficient (CLED) agar. Colonies with a significant number (10^5^ CFU/ml) from CLED agar were subjected to Gram staining and then sub-cultured on MacConkey (Gram negative) and Blood agar plates (gram positive) (BIO MARK Laboratories, India) for identification. Cultures were incubated at 37^0^c for 24 hours. After a series of biochemical tests were performed to identify Gram-negative isolates (performed using Triple sugar iron agar, Urea agar, Citrate agar, Lysine iron agar, Motility medium and Indole test) and Gram-positive isolates (Catalase, coagulase, bile-esculin hydrolysis, and optochin sensitivity). Then, the Kirby-Bauer disk-diffusion method of AST commences on Muller-Hinton agar (BIO MARK Laboratories, India) to determine its susceptibility to antimicrobial agents by incubating at 37^0^c for 18 hours. Antimicrobial discs used were ampicillin (10 μg), amoxicillin-clavulanic acid (20/10 μg), cefoxitin (30 μg), ciprofloxacin (5 μg), gentamycin (10 μg), nitrofurantoin (300 μg), norfloxacin (10 μg), amikacin (30 μg), kanamycin (30 μg), tetracycline (30 μg), tobramycin(10 μg), ceftriaxone (30 μg), nalidixic acid (30 μg), cefuroxime (30 μg), cefotaxime (30 μg), ceftazidime (30 μg), vancomycin(30 μg), meropenem (10 μg), Trimethoprim-Sulfamethoxazole (1.25/23.75 μg), chloramphenicol (30 μg), and penicillin (10 units) (HI Media Laboratories, India). The AST results were collected, and multidrug-resistant (MDR) isolates were identified. Multidrug resistance is the in vitro non-susceptibility to at least one drug in more than two classes of antimicrobial agents [17]. The antimicrobial discs were selected, and AST results were interpreted, based on the clinical laboratory standards institute (CLSI) guideline [18].

The data were summarized and entered into a statistical package for social sciences (SPSS) version 23 software and were analyzed using the software (SPSS) for descriptive statistics. Then, the descriptive results were presented with tables and graphs. The trend of antimicrobial resistance was determined by dividing the number of resistant isolates to the total isolates tested in each year. The data were collected by investigators with data quality and completeness checks throughout the collection period, at the end of data collection, and after entry to SPSS for statistical analysis.

## Results

### Socio-demographic characteristics and rate of infection

From Jan 2010–2019, the UoGCSH bacteriology laboratory analyzed 4441 urine samples from UTI suspected patients. Of those patients, 54.8% were females. The age group 21–30 years accounts for the highest proportion (27.1%) of UTI suspected patients, while the highest prevalence of UTI (47.4%) falls in the age group 61–70 years, and the least affected (15.4%) falls in the age group 2–10 years **(Table 1**).

**Table 1:** The distribution of uropathogenic isolates with sex and age at the university of Gondar comprehensive specialized hospital, 2010–2019.

Variable	Category of variable	Frequency (%)	UTI (significant bacteriuria)	
			Positive N (%)	Negative N (%)
Sex	Male	2006(45.2)	494(24.6)	1512(75.4)
	Female	2435(54.8)	578(23.7)	1857(76.3)
Age	≤ One year	307(6.9)	64(20.8)	243(79.2)
	2–10 years	702(15.8)	108(15.4)	594(84.6)
	11–20 years	555(12.5)	109(19.6)	446(80.4)
	21–30 years	1205(27.1)	268(22.2)	937(77.8)
	31–40 years	554(12.5)	123(22.2)	431(77.8)
	41–50 years	384(8.6)	97(25.3)	287(74.7)
	51–60 years	285(6.4)	96(33.7)	189(66.3)
	61–70 years	228(5.1)	108(47.4)	120(52.6)
	71 and above	221(5)	99(44.8)	122(55.2)
	Total	4441(100)	1072(24.1)	3369(75.9)

Key: Frequency (%) column is calculated from the total sample size (4441); UTI rate of each category of variables is calculated row wise.

### The proportion of uropathogenic bacterial isolates

A total of 1072 (24.1%) significant bacteriuria of monomorphic bacterial growth was recorded. Of these isolates, 879 (82%) were Gram-negative bacteria. *Escherichia coli* (37.7%) and *K*. *pneumoniae* (11.4%) were the predominant of all isolates while *S*. *aureus* (9.14%) was the leading Gram-positive and the third most common of all uropathogenic isolates in this study **(Table 2**).

**Table 2:** The proportion of uropathogenic isolates at the university of Gondar comprehensive specialized hospital, 2010–2019.

Species of isolates	Frequency	Percentage (from total UTI patients; 1072)
*E*. *coli*	404	37.7
*K*. *pneumoniae*	122	11.4
CONS	46	4.3
*S*.*aureus*	98	9.14
*Prouteus* Spp.	17	1.6
*Citrobacter* Spp	76	7.1
*Salmonella* Spp	6	0.6
GNR	111	10.34
*Enterobacter* Spp	37	3.45
*Streptococcus* Spp	26	2.42
*Klebsiella* Spp	36	3.35
*Pseudomonas* Spp	6	0.55
*Providencia* Spp	5	0.46
*K*.*ozaenae*	45	4.2
*Shigella* Spp	8	0.74
*Enterococcus* Spp	23	2.14
*Serratia* Spp	4	0.37
*M*.*morgani*	2	0.18

Key: CONS- Coagulase Negative Staphylococci, GNR-Gram negative rods, Spp- Species.

### Antimicrobial resistance pattern of Gram-positive isolates

Gram-positive isolates showed a high resistance to tetracycline (84.8%) and penicillin (71.6%). Antimicrobial agents most effective against Gram-positive uropathogens were vancomycin and nitrofurantoin. *Staphylococcus aureus* was the most common isolate comprising about 51% of Gram-positive isolates. It was highly resistant to tetracycline (85.7%) and trimethoprim-sulfamethoxazole (83.5%). In addition, 30% of *S*. *aureus* were resistant to cefoxitin **(Table 3)**.

**Table 3:** The proportion of resistant Gram-positive isolates among UTI patients at the University of Gondar comprehensive specialized hospital, 2010–2019.

Antibiotics	CONs N (%)	*S*. *aureus*	*Streptococcus* spp N (%)	*Enterococcus* spp N (%)	Row Total N (%)
AMP	ND	ND	4/8(50)	7/7(100)	11/15(73.3)
PEN	16/19(84.2)	22/32(68.8)	7/12(58.3)	3/4(75)	48/67(71.6)
CIP	13/20(65)	36/57(63.2)	7/15(46.7)	12/20(60)	68/112(60.7)
GEN	9/24(37.5)	13/30(43.3)	7/11(63.6)	4/7(57.1)	33/72(45.8)
NIT	2/5(40)	3/21(14.3)	1/10(10)	6/12(50)	12/48(25)
NOR	11/15(73.3)	24/39(61.5)	4/6(66.7)	5/7(71.4)	44/67(65.7)
TET	22/26(84.6)	48/56(85.7)	9/12(75)	5/5(100)	84/99(84.8)
CAF	9/18(50)	11/29(37.9)	0/9(0)	1/6(16.7)	21/62(33.9)
FOX	1/1(100)	3/10(30)	1/4(25)	2/2(100)	7/17(41.2)
SXT	17/19(89.5)	33/41(80.5)	12/13(92.3)	9/12(75)	71/85 (83.5)
CRO	9/25(36)	11/35(31.4)	6/11(54.5)	2/4(50)	28/75(37.3)
VAN	ND	ND	1/8(12.5)	2/6(33.3)	3/14(21.4)

Key: AMP-ampicillin, PEN- penicillin, AMC-amoxicillin-clavulanic acid, CIP- ciprofloxacin, GEN-gentamycin, NIT- nitrofurantoin, NOR- norfloxacin, TET- tetracycline, TOB- tobramycin, FOX- cefoxitin, CRO- ceftriaxone, VAN-vancomycin, NA- nalidixic acid, OXA-oxacillin, ND-not done.

### Antimicrobial resistance pattern of Gram-negative isolates

Gram-negative isolates showed a high resistance rate to ampicillin (92.5%), amoxicillin-clavulanate (80.1%), tetracycline (79.3%), cefuroxime (79.2%) and trimethoprim-sulfamethoxazole (78.3%). Their resistance against cephalosporin drugs ranges from 50%-79.2%, while fluoroquinolone resistance was 51.5% - 66.5%. The least resistance was reported against amikacin (20%) and meropenem (26.4%). *Escherichia coli*, which accounted for 45.9% of Gram-negative isolates showed 88.9%, 83.6%, 76.5%, and 74% resistance to ampicillin, tetracycline, trimethoprim-sulfamethoxazole, and amoxicillin-clavulanate, respectively. All Gram-negative isolates had a high ampicillin resistance ranging from 89–100% **(Table 4)**.

**Table 4:** The proportion of resistant Gram-negative isolates among UTI patients at the University of Gondar comprehensive specialized hospital, 2010–2019.

Antibiotics	*E*. *coli* N (%)	*K*.*pneu moniae* N (%)	*Proteu s* spp N (%)	*Citrobacter* spp N (%)	*Salmon ella* spp N(%)	NLFGNR N (%)	*Enterob acter* spp N (%)	*Klebsiella* spp N (%)	LFGNR N (%)	*Pseudo monas* spp N (%)	*Provid encia* spp N (%)	*K*.*ozaena e* N (%)	*Shigell a* spp N (%)	*Serratia* spp N (%)	Row Total N (%)
AMP	219/246 (88.9)	64/65 (98.5)	12/13 (92)	48/49 (98)	3/4(75)	32/35 (91.4)	19/20(95.0)	27/27(100)	26/29(89.6)	ND	2/2(100)	21/22(95.5)	4/4(100)	2/2(100)	479/518 (92.5)
AMC	128/173 (74)	44/51 (86.3)	5/9 (55.6)	31/32 (96.9)	3/3(100)	20/27 (74)	9/10(90.0)	15/16(93.8)	16/21(76.2)	ND	2/2(100)	17/19(89.5)	2/2(100)	1/1(100)	293/366(80.1)
CIP	155/279 (55.6)	30/78 (38.5)	8/15 (53.3)	29/57 (50.9)	1/4(25)	20/44 (45.5)	13/20(65)	15/27(55.6)	15/32(46.9)	1/6(16.7)	2/5(40)	15/26(57.7)	5/7(71.4)	1/2(50)	309/602 (51.3)
NOR	122/179 (61.9)	19/48 (40)	4/7 (57.1)	14/34 (41.2)	1/2(50)	18/33 (54.5)	7/13(53.8)	7/17(41.2)	17/26(65.4)	1/4(25)	2/5(40)	14/20(70)	3/5(60)	1/2(50)	230/395(58.2)
NA	45/69 (65.2)	18/29 (66.7)	1/1 (100)	9/14 (64.3)	ND	7/12 (58.3)	3/8(37.5)	8/11(72.7)	10/13(76.9)	ND	ND	7/11(63.6)	1/1(100)	1/1(100)	110/170(64.7)
TOB	33/72 (45.8)	18/29 (62.1)	1/5 (20)	7/14 (50)	ND	6/9 (66.7)	3/4(75)	1/4(25)	2/7(28.6)	1/1(100)	ND	2/9(22.2)	1/3(33.3)	0/1	75/158(47.5)
GEN	103/240 (42.9)	35/58 (58.6)	7/13 (53.8)	20/36 (55.6)	2/3(66.7)	19/30 (63.3)	14/17(82.4)	8/15(53.3)	16/29(55.2)	1/4(25)	2/4(50)	15/22(68.2)	3/5(60)	ND	245/476(51.5)
AMK	6/25(24)	4/11 (36.4)	ND	0/11	ND	2/3 (66.7)	0/5	0/2	0/3	0/1	ND	1/4(25)	ND	ND	13/65(20)
KAN	7/13(53.8)	5/7 (71.4)	ND	1/1 (100)	1/1(100)	1/1 (100)	1/1(100)	ND	1/2(50)	ND	ND	1/2(50)	1/4(25)	ND	19/32(59.4)
NIT	21/133 (15.8)	13/41 (31.7)	4/4 (100)	11/21 (52.4)	ND	12/24 (50)	2/7(28.6)	6/7(85.7)	5/17(29.4)	3/3(100)	ND	0/11	0/1	1/1 (100)	78/270(28.9)
SXT	186/243 (76.5)	55/66 (83.3)	8/10 (80)	29/37 (78.4)	2/3(66.7)	33/38 (86.8)	15/17(88.2)	16/20(80)	15/21(71.4)	3/5(60)	3/3(100)	24/29(82.8)	3/7(42.9)	1/3 (33.3)	393/502(78.3)
TET	163/195 (83.6)	33/46 (71.7)	10/11 (91)	31/40 (77.5)	0/1	24/29 (82.8)	14/18(77.8)	16/18(88.9)	19/24(79.2)	2/3(66.7)	4/5(80)	12/18(66.7)	2/5(40)	2/2 (100)	332/415(79.3)
CAF	43/163 (26.4)	22/38 (57.9)	8/10 (80)	17/32 (53.1)	1/2(50)	12/20 (60)	9/15(60)	5/13(38.5)	9/18(50)	2/2(100)	1/4(25)	9/15(60)	2/5(40)	1/1 (100)	141/338(41.7)
CRX	26/38 (68.4)	15/17 (88.2)	1/3 (33.3)	5/5 (100)	1/1(100)	6/6 (100)	3/3(100)	2/2 (100)	4/6(66.7)	1/1(100)	ND	8/9(88.9)	1/1(100)	ND	73/92(79.3)
CRO	93/205 (45.4)	33/54 (61.1)	5/14 (35.7)	27/41 (65.9)	2/3(66.7)	18/27 (66.7)	9/11(81.8)	17/24(71)	14/22(63.6)	1/2(50)	2/4(50)	15/17(88.2)	3/6(50)	1/2(50)	240/432(55.6)
FOX	6/18(33.3)	¾ (75)	ND	3/5 (60)	ND	1/1(100)	1/2(50)	2/3(66.7)	2/3(66.7)	1/1(100)	ND	1/2(50)	ND	ND	20/39(51.3)
CTX	4/10(40)	4/4 (100)	ND	0/4	1/1(100)	0/1(0)	2/3(66.7)	0/1	2/2(100)	ND	ND	ND	ND	ND	13/26(50)
CAZ	14/25 (56)	6/10 (60)	0/2	1/4 (25)	ND	3/3 (100)	2/3(66.7)	1/3(33.3)	3/6(50)	ND	ND	5/5(100)	0/1	ND	35/62(56.5)
MER	9/34 (26.5)	3/17 (17.6)	0/2	2/7 (28.6)	ND	4/9 (44.4)	4/5(80)	0/2	0 /3	ND	ND	2/9(22.2)	0/3	ND	24/91(26.4)

Key: AMP-ampicillin, AMC-amoxicillin-clavulanic acid, CIP- ciprofloxacin, GEN-gentamycin, NIT- nitrofurantoin, NOR- norfloxacin, AMK-amikacin, KAN-Kanamycin, TET- tetracycline, TOB- tobramycin, FOX- cefoxitin, CRO- ceftriaxone, NA- nalidixic acid, CRX-cefuroxime, CTX-cefotaxime, CAZ-ceftazidime, MER- meropenem, SXT-Trimethoprim-Sulfamethoxazole, CAF-chloramphenicol, LFGNR-Lactose fermenting Gram negative rods, NLFGNR- None lactose fermenting Gram negative rods, ND-not done, N- Number of organisms of a specified species tested to each antimicrobial agent. Numerators represent number of resistant bacteria to each antimicrobial while the denominators are the number of bacteria tested against each antimicrobial agent (both resistant and susceptible).

### The yearly basis of resistance pattern of the classes of antimicrobial agents

The resistance rate of isolated bacteria was seen to be inconsistent to the majority of the tested antimicrobial classes. However, the resistance to cephalosporins has been rising, particularly from 2014 to 2019 (Table 5).

**Table 5:** Yearly basis of antimicrobial resistance to classes of antimicrobial agents, 2010–2019.

	Number N (%) of isolates tested to each class of antibiotics										
Class	2010	2011	2012	2013	2014	2015	2016	2017	2018	2019	Total
Penicillin	133/144 (92.4)	98/114(86)	148/175 (84.6)	109/119 (91.6)	81/89 (91)	71/79(90)	35/43(81.4)	42/48(87.5)	75/97(77.3)	49/58(84.5)	841/966 (87.1)
Fluoroquinolone	97/177 (54.8)	67/125 (53.6)	104/174 (59.8)	92/165(55.8)	78/126(61.9)	42/87(48.3)	29/65(44.6)	83/133(62.4)	93/160(58.1)	80/129(62)	765/1341(57)
Aminoglycoside	54/100(54)	29/58 (50)	10/23 (43.5)	39/61(63.9)	33/68(48.5)	30/63(47.6)	14/24(58.3)	26/52(50)	93/222(41.9)	57/122(46.7)	385/793(48.5)
Cephalosporin	44/56(78.6)	32/43 (74.4)	27/56(48.2)	54/94(57.4)	34/76 (44.7)	30/56(53.6)	14/25(56)	11/19(57.9)	90/127(70.9)	80/113(70.8)	416/665(62.6)
Folate pathway inhibitor (SXT)	80/95 (84.2)	51/69 (73.9)	15/22 (68.2)	26/37(70.3)	35/42 (83.3)	37/53 (69.8)	30/36 (83.3)	60/69(87)	62/79 (78.5)	68/85 (80)	464/587(79)
NIT	ND	ND	ND	ND	9/29 (31)	9/49 (18.4)	4/28 (14.3)	8/40 (20)	37/109(33.9)	23/63 (26.7)	90/318(28.3)
TET	79/102(77.5)	61/77 (79.2)	67/81 (82.7)	76/91(83.5)	39/49 (79.6)	49/56(87.5)	21/29 (72.4)	5/7 (71.4)	13/15 (86.7)	6/7 (85.7)	416/514(80.9)
CAF	41/97 (42.3)	26/66 (39.4)	7/23 (30.4)	27/43(62.8)	25/78 (32.1)	12/29(41.4)	3/15 (20)	12/31(38.7)	5/10 (50)	4/8 (50)	162/400(40.5)
Carbapenem	ND	ND	ND	ND	ND	ND	ND	ND	13/53 (20.8)	11/38 (28.9)	24/91 (26.4)
Glycopeptide (VAN)	ND	ND	ND	ND	ND	ND	ND	ND	2/9 (22.2)	1/5 (20)	3/14 (21.4)

ND- Antimicrobial susceptibility test was not done, VAN- vancomycin.

### Antimicrobial resistance (AMR) trend and multidrug resistance (MDR) rate of isolates

**Fig 1:**
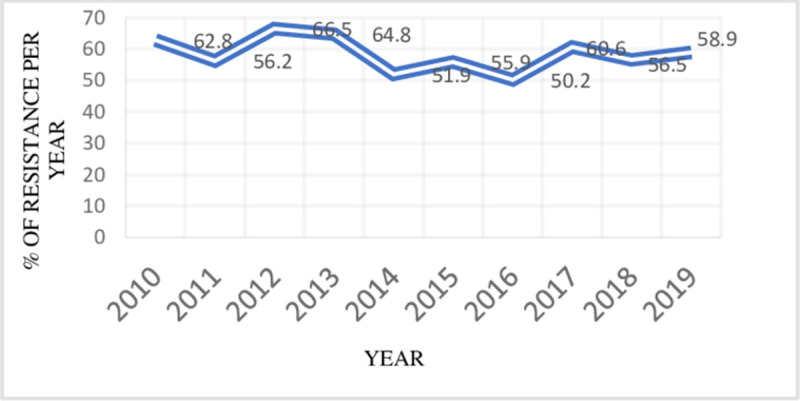
The overall trend of antimicrobial resistance of isolates to the tested antibiotics among UTI patients at the University of Gondar comprehensive specialized Hospital, 2010–2019.

In the last ten consecutive years (2010–2019), the antimicrobial resistance trend of uropathogens ranges from 50 to 66.5%. The resistance rate was highest (66.5%) in 2012, and the lowest (50.2%) observed resistance was in 2016. From 2012 to 2016, there was a reduction in antibiotic resistance rates. However, there was a slight increment in the resistance rate from 2016 to 2019 **(Fig 1**).

**Fig 2:**
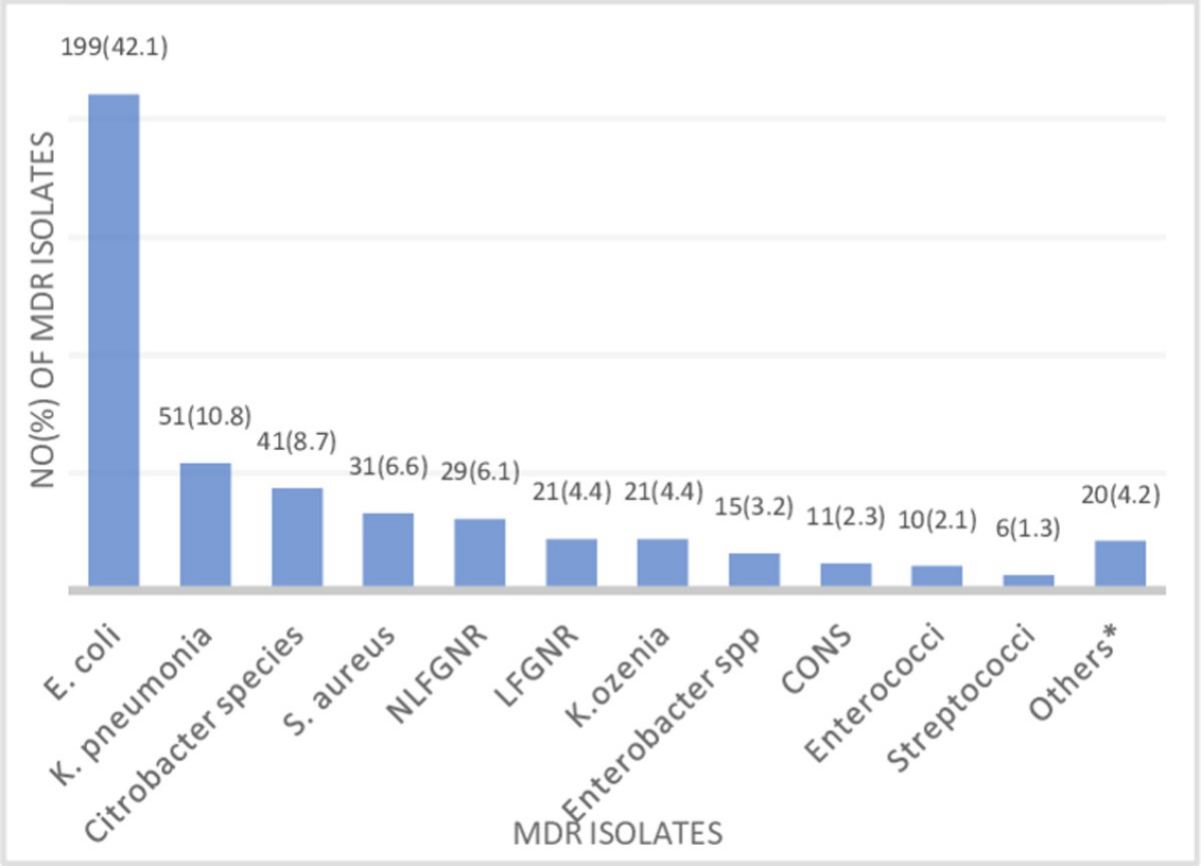
Frequency of MDR species isolated in 10 years retrospective study (473/1072 = 44.1%). Key: Proteus 7(1.5%), shigella 5(1.1%), Providencia 3(0.6%), Pseudomonas 2(0.4%), Salmonella, Serratia, *M*. *morganii* 1(0.2%) each.

Multidrug-resistant (MDR) isolates were 473 (44.1%) of the total 1072 uropathogens. *E*. *coli* 199 (42.1%) and *K*. *pneumonia* 51(10.8%) were the predominant MDR uropathogens, which together account for more than half of the total MDR isolates. Among Gram-positive uropathogens, *S*. *aureus* 31(6.6%) was the leading MDR isolate, placed 4^th^ among all MDR isolates **(Fig 2)**.

## Discussion

Globally, human health is in danger from antimicrobial-resistant infection. To strengthen knowledge of AMR through surveillance and research World Health Organization (WHO) opened a program called the Global Antimicrobial Resistance Surveillance System (GLASS). *Escherichia coli*, *K*. *pneumoniae*, and *S*. *aureus*, which are the leading etiologic agents of UTI and common resistant bacteria, are among the GLASS targets [19]. Epidemiological studies with time in different geographic regions are the first and vital steps for selecting effective antimicrobial agents for treatment, preventive and control actions [20]. The overall prevalence of UTI in this study was 1072(24.1%) [95% CI, (22.9–25.4)], with a proportional infection rate in males (24.6%) and females (23.7%). This finding is consistent with results from studies conducted in Dessie (22.7%) [21] and Addis Ababa, Ethiopia (23.32%) [22], as well as other parts of the world such as India (22.8%) [23] and central Europe (26.9%) [24]. The age-wise distribution of the infection reveals 47.4% UTI in the elderly population above 60 years. In contrast, according to a study done in Addis Ababa the most affected age group of participants was the age group 21–30 years [22]. Similar to our study, an increased infection rate in the elderly participants has been reported elsewhere [16]. The elderly population might be at risk of acquiring UTI due to age-related weakened immunity, change in vaginal hormonal secretion, or other comorbidities [25]. On the other hand, the rate of UTI in our study was lower than studies conducted in Bahir Dar, Ethiopia (30.5%) [26], India (38.84%) [27], India ((28.2%) [28], Sudan (91%) [29], but higher than the prevalence studies conducted in Iran (15%) [20] and India (15.9%) [30]. Geographic and population differences, study design, or the laboratory method used among studies may explain the difference in the prevalence rate.

Regarding the proportion of bacterial isolates in this study, *E*. *coli* 404 (37.7%) was the leading isolate among uropathogens, followed by *K*. *pneumoniae* (11.4%) and *S*. *aureus* (9.14%). Similar findings have been reported in different geographic regions; Gondar [16], Bahir Dar [26] and Hawassa [31], and Addis Ababa, Ethiopia [27], and India [28].

The resistance rate of Gram-positive isolates was high to agents such as tetracycline (84.8%), trimethoprim-sulfamethoxazole (83.5%), fluoroquinolones (60.7–65.7%), and penicillin (71.6%). This figure is much higher than the resistance rate (34.6%) reported in Iran [20]. Moreover, more than 75% of Gram-negative isolates in this study, were resistant to ampicillin (92.3%), amoxicillin-clavulanate (80.1%), tetracycline (79.3%), cefuroxime (79.2%), and Trimethoprim-sulfamethoxazole (78.3%). *Escherichia coli* isolates were highly resistant to ampicillin (88.9%), tetracycline (83.6%), trimethoprim-sulfamethoxazole (76.5%), and amoxicillin-clavulanate (74%). The resistance rate continues to grow even for those antimicrobial agents including cefuroxime (68.4%) which are limited to selected tertiary hospitals. Comparable resistance rate among isolates were reported in Sudan; ampicillin (94%), amoxicillin-clavulanate (90%), tetracycline (76%), norfloxacin (74%), trimethoprim-sulfamethoxazole (88%) and ceftriaxone (68%) [31]. In this study, more than half of the Gram negative isolates were resistant to fluoroquinolones which outnumbers a report in the USA (24.3%-25.8%) [32]. Moreover, nitrofurantoin (35%), ciprofloxacin (28.8%), and ceftriaxone (25.9%) were better agents for uropathogens in another study [33].

In this study, 26.4% and 20% of Gram-negative isolates were resistant to meropenem and amikacin respectively. It is worrisome because these agents were considered the most effective agents in treating UTIs [32]. Furthermore, the rate of resistance of uropathogens to amikacin (20%), ciprofloxacin (51.3%), and cefuroxime (79.3%) in this study were lower than the resistance rate reported in Turkey [34]. Different reports showed that uropathogens are highly resistant to ampicillin, amoxicillin-clavulanate, and trimethoprim-sulfamethoxazole [33, 34]. The recommendations from national guidelines for antimicrobial use in different countries could have resulted in varying resistance among countries [35]. The resistance trend of uropathogenic isolates over ten consecutive years (January 2010–2019) ranges from 50.2% in 2016 to 66.5% in 2012. The resistance rate of isolated bacteria to antimicrobial classes was inconsistent but the resistance to cephalosporins has shown increasing pattern particularly from 2014 to 2019. Absence of uniform supply and hence, irregular use of these antibiotics in the laboratory can be mentioned for such inconsistent trend of resistance. The rising resistance to cephalosporins may be due to the rising preference and clinical use of this agent.

This study showed an inconsistent trend of the overall resistance rate with a reducing rate between 2012 and 2016, while a slight increment from 2016 to 2019. In general, the overall resistance rate surpasses 50% over the study period (**Fig 1**). Moreover, there were vancomycin-resistant *Enterococcus* species (33.3%) in this study, which was a higher prevalence than a report (14.8%) by Melese et al. in Ethiopia [36] but lower than 54% resistance reported in Sudan [29]. In addition, 6(50%) nitrofurantoin resistant *Enterococcus* species were isolated in this study which was higher than (9.8%) vancomycin and (0–40%) nitrofurantoin resistant *Enterococcus* species reported in England [37]. Methicillin-resistant *S*. *aureus* (MRSA) is an emerging threat evolving rapidly, and 30% of MRSA strains were isolated in this study which needs an urgent response [38].

Multidrug resistance is a concern of the medical community because there is a run out of effective antimicrobial agents to relieve the suffering of patients and save lives [39]. We found a total of 473(44.1%) MDR UTI which concords to a result reported in Tunisia (45.1%) [40]. However, our finding was higher than 25% in Portugal [41] and 36.5% in Germany [42]. The result of this study otherwise, was lower compared to results from Hawassa, southern Ethiopia (80.3%) [31], in Serra Leone (85.7%) [43], Saudi Arabia (80%) [1] and Serbia (53.8%) [44]. The Geographic variation, limited activity towards implementation of antimicrobial stewardship program, and a different definition of MDR might contribute to the observed differences. The species *E*. *coli* (42.1%), *K*. *pneumonia* (10.8%), *Citrobacter* species (8.7%), and *S*. *aureus* (6.6%) were the most common MDR isolates in this study. This result was lower than the 79.3% MDR in southern Ethiopia among HIV patients [45], who frequently take antibiotics and are at higher risk of MDR infections. In general, MDR, Carbapenem resistance, MRSA, and vancomycin resistance have been observed in this study. Hence, healthcare professionals and other stakeholders need to be curious about the supply and control of antimicrobial agents as we are on the verge of loss of effective agents [20]. Considering the existing resistance in the hospital from our result, healthcare providers in the hospital would selectively use the effective antibiotics. The global community would understand the burden of resistance in the area and design comprehensive policies by aggregating with reports from diverse geographic settings. Limitation, as the study was a retrospective study, we did not analyze factors contributing to resistance. The resistance trend of each species was not determined because of inconsistent use of antimicrobials to each species. In addition, it is a single-center hospital-based study among symptomatic patients. We were also not able to differentiate between inpatients and out patients and compare the rate of UTI. As the laboratory did not have any molecular based detection, this study was limited to conventional culture-based results.

## Conclusions

The overall of prevalence of UTI was high and elderly patients were most affected. *Escherichia coli* was the most common bacterial uropathogen, followed by *K*. *pneumonia* and *S*. *aureus*. Multidrug resistance in this study is high, which alarms a need for problem resolution using routine diagnosis and antimicrobial susceptibility testing rather than empirical treatment. A continuous revision of UTI treatment guideline to replace ineffective agents with potent alternatives should be done. A prospective multicenter study including asymptomatic population is indispensable to know status of resistance.

## List of abbreviations

AMR: Antimicrobial resistance
AST: Antimicrobial Susceptibility Test
CLED: Cysteine Lactose Electrolyte Deficient
CLSI: Clinical Laboratory Standards Institute
CMHS: Collage of Medicine and Health Science
MDR: Multidrug Resistance
MRSA: Methicillin resistant *staphylococcus aureus*
SBLS: School of Biomedical and Laboratory Science
UoGCSH: University of Gondar Comprehensive Specialized Hospital
UTI: Urinary tract infection

## References

[1] Ahmed SS, Shariq A, Alsalloom AA, Babikir IH, Alhomoud BN. Uropathogens and their antimicrobial resistance patterns: Relationship with urinary tract infections. *Int J Health Sci* (Qassim). 2019;13(2):48–55. 30983946 PMC6436442

[2] Stamm WE, Hooton TM. Management of urinary tract infections in adults. *New England journal of medicine*. 1993;329(18):1328–34. doi: 10.1056/NEJM199310283291808 8413414

[3] Bennett JE, Dolin R, Blaser MJ, Mandell GL. Mandell, Douglas, and bennett’s principles and practice of infectious diseases E-Book: Elsevier Health Sciences; 2009.

[4] Bitew A, Molalign T, Chanie M. Species distribution and antibiotic susceptibility profile of bacterial uropathogens among patients complaining urinary tract infections. *BMC infectious diseases*. 2017;17(1):654. doi: 10.1186/s12879-017-2743-8 28962545 PMC5622472

[5] Flores-Mireles AL, Walker JN, Caparon M, Hultgren SJ. Urinary tract infections: epidemiology, mechanisms of infection and treatment options. *Nature reviews microbiology*. 2015;13(5):269–84. doi: 10.1038/nrmicro3432 25853778 PMC4457377

[6] Ronald A. The etiology of urinary tract infection: traditional and emerging pathogens. T*he American journal of medicine*. 2002;113(1):14–9. doi: 10.1016/s0002-9343(02)01055-0 12113867

[7] Nguyen H, Tanagho E, McAninch J. Smith’s general urology. 2004.

[8] Schaeffer A, Rajan N, Cao Q, Anderson B, Pruden DL, Sensibar J, et al. Host pathogenesis in urinary tract infections. *International journal of antimicrobial agents*. 2001;17(4):245–51. doi: 10.1016/s0924-8579(01)00302-8 11295403

[9] Foxman B. Epidemiology of urinary tract infections: incidence, morbidity, and economic costs. *The American journal of medicine*. 2002;113(1):5–13. 10.1016/s0002-9343(02)01054-9 12113866

[10] Organization WH. WHO report on surveillance of antibiotic consumption: 2016–2018 early implementation. 2018.

[11] Khameneh ZR, Afshar AT. Antimicrobial susceptibility pattern of urinary tract pathogens. *Saudi Journal of Kidney Diseases and Transplantation*. 2009;20(2):251. 19237813

[12] Alemu A, Dagnew M, Alem M, Gizachew M. Uropathogenic bacterial isolates and their antimicrobial susceptibility patterns among HIV/AIDS patients attending Gondar University Specialized Hospital, Gondar, Northwest Ethiopia. *Journal of Microbiology Research and Reviews*. 2013;1(4):42–51.

[13] Goossens H, Ferech M, Vander Stichele R, Elseviers M, Group EP. Outpatient antibiotic use in Europe and association with resistance: a cross-national database study. *The Lancet*. 2005;365(9459):579–87. doi: 10.1016/S0140-6736(05)17907-0 15708101

[14] O’Neill J. Review on antimicrobial resistance. Antimicrobial resistance: tackling a crisis for the health and wealth of nations. 2014;2014(4).

[15] Ashkenazi S, Even-Tov S, Samra Z, Dinari G. Uropathogens of various childhood populations and their antibiotic susceptibility. *The Pediatric infectious disease journal*. 1991;10(10):742–6. doi: 10.1097/00006454-199110000-00005 1945576

[16] Tiruneh M, Yifru S, Gizachew M, Molla K, Belyhun Y, Moges F, et al. Changing trends in prevalence and antibiotics resistance of uropathogens in patients attending the Gondar University Hospital, Northwest Ethiopia. *International journal of bacteriology*. 2014;2014:629424. doi: 10.1155/2014/629424 26904737 PMC4745455

[17] Magiorakos A-P, Srinivasan A, Carey Rt, Carmeli Y, Falagas Mt, Giske Ct, et al. Multidrug-resistant, extensively drug-resistant and pandrug-resistant bacteria: an international expert proposal for interim standard definitions for acquired resistance. *Clinical microbiology and infection*. 2012;18(3):268–81. doi: 10.1111/j.1469-0691.2011.03570.x 21793988

[18] Clinical, Institute LS. Performance standards for antimicrobial susceptibility testing. Clinical and Laboratory Standards Institute Wayne, PA; 2017.

[19] WHO. Worldwide country situation analysis: response to antimicrobial resistance. World Health Organization Geneva; 2015.

[20] Mihankhah A, Khoshbakht R, Raeisi M, Raeisi V. Prevalence and antibiotic resistance pattern of bacteria isolated from urinary tract infections in Northern Iran. *Journal of research in medical sciences*: the official journal of Isfahan University of Medical Sciences. 2017;22:108. doi: 10.4103/jrms.JRMS_889_16 29026424 PMC5629843

[21] Kibret M, Abera B. Prevalence and antibiogram of bacterial isolates from urinary tract infections at Dessie Health Research Laboratory, Ethiopia. *Asian Pacific journal of tropical biomedicine*. 2014;4(2):164–8. doi: 10.1016/S2221-1691(14)60226-4 25182289 PMC3819486

[22] Kabew G, Abebe T, Miheret A. A retrospective study on prevalence and antimicrobial susceptibility patterns of bacterial isolates from urinary tract infections in Tikur Anbessa Specialized Teaching Hospital Addis Ababa, Ethiopia, 2011. *Ethiopian Journal of Health Development*. 2013;27(2):111–7.

[23] Murugan K, Savitha T, Vasanthi S. Retrospective study of antibiotic resistance among uropathogens from rural teaching hospital, Tamilnadu, India. *Asian pacific Journal of tropical Disease*. 2012;2(5):375–80.

[24] Hrbacek J, Cermak P, Zachoval R. Current antibiotic resistance trends of uropathogens in Central Europe: Survey from a Tertiary hospital urology department 2011–2019. *Antibiotics*. 2020;9(9):630. doi: 10.3390/antibiotics9090630 32971752 PMC7559630

[25] Storme O, Tiran Saucedo J, Garcia-Mora A, Dehesa-Dávila M, Naber KG. Risk factors and predisposing conditions for urinary tract infection. *Therapeutic advances in urology*. 2019;11:1756287218814382. doi: 10.1177/1756287218814382 31105772 PMC6502981

[26] Derbie A, Hailu D, Mekonnen D, Abera B, Yitayew G. Antibiogram profile of uropathogens isolated at Bahir Dar regional health research laboratory centre, northwest Ethiopia. *The Pan African Medical Journal*. 2017;26:134. doi: 10.11604/pamj.2017.26.134.7827 28533857 PMC5429430

[27] Somashekara SC, Deepalaxmi S, Jagannath N, Ramesh B, Laveesh MR, Govindadas D. Retrospective analysis of antibiotic resistance pattern to urinary pathogens in a Tertiary Care Hospital in South India. *Journal of basic and clinical pharmacy*. 2014;5(4):105–108. doi: 10.4103/0976-0105.141948 25316990 PMC4194940

[28] Kalal BS, Nagaraj S. Urinary tract infections: a retrospective, descriptive study of causative organisms and antimicrobial pattern of samples received for culture, from a tertiary care setting. *Germs*. 2016;6(4):132–138 doi: 10.11599/germs.2016.1100 28053916 PMC5187754

[29] Saad D, Gameel S, Ahmed S, Basha E, Osman M, Khalil E. Etiological Agents of Urinary Tract Infection and 7 Years Trend of Antibiotic Resistance of Bacterial Uropathogens in Sudan. *The Open Microbiology Journal*. 2020;14(1):312–320.

[30] Ramu S. Retrospective study on susceptibility and resistance pattern of urinary pathogens in a tertiary care hospital. *International Journal of Basic & Clinical Pharmacology*. 2019;8(10):2211–2215.

[31] Mechal T, Hussen S, Desta M. Bacterial Profile, Antibiotic Susceptibility Pattern and Associated Factors Among Patients Attending Adult OPD at Hawassa University Comprehensive Specialized Hospital, Hawassa, Ethiopia. *Infection and Drug Resistance*. 2021;14:99–110. doi: 10.2147/IDR.S287374 33469325 PMC7813457

[32] Critchley IA, Cotroneo N, Pucci MJ, Mendes R. The burden of antimicrobial resistance among urinary tract isolates of Escherichia coli in the United States in 2017. *PloS one*. 2019;14(12):e0220265. doi: 10.1371/journal.pone.0220265 31821338 PMC6903708

[33] Belete MA, Saravanan M. A systematic review on drug resistant urinary tract infection among pregnant women in developing countries in Africa and Asia; 2005–2016. *Infection and drug resistance*. 2020;13:1465–77. doi: 10.2147/IDR.S250654 32547115 PMC7245001

[34] Gunduz S, Altun HU. Antibiotic resistance patterns of urinary tract pathogens in Turkish children. *Global health research and policy*. 2018;3(1):1–5. doi: 10.1186/s41256-018-0063-1 29568806 PMC5856228

[35] Food E. Medicine and Healthcare Administration and Control Authority. Continuing Professional Development (CPD) Guideline for Health Professionals in Ethiopia Addis Ababa: FMoH. 2013.

[36] Melese A, Genet C, Andualem T. Prevalence of Vancomycin resistant enterococci (VRE) in Ethiopia: a systematic review and meta-analysis. BMC infectious diseases. 2020;20(1):1–12. doi: 10.1186/s12879-020-4833-2 32046668 PMC7014939

[37] Toner L, Papa N, Aliyu SH, Dev H, Lawrentschuk N, Al-Hayek S. Vancomycin resistant enterococci in urine cultures: Antibiotic susceptibility trends over a decade at a tertiary hospital in the United Kingdom. *Investigative and clinical urology*. 2016;57(2):129–134. doi: 10.4111/icu.2016.57.2.129 26981595 PMC4791667

[38] Koch G, Yepes A, Förstner KU, Wermser C, Stengel ST, Modamio J, et al. Evolution of resistance to a last-resort antibiotic in Staphylococcus aureus via bacterial competition. *Cell*. 2014;158(5):1060–71. doi: 10.1016/j.cell.2014.06.046 25171407 PMC4163622

[39] Gajdács M, Burián K, Terhes G. Resistance levels and epidemiology of non-fermenting gram-negative bacteria in urinary tract infections of inpatients and outpatients (RENFUTI): a 10-year epidemiological snapshot. *Antibiotics*. 2019;8(3):143. doi: 10.3390/antibiotics8030143 31505817 PMC6784256

[40] Ben Ayed H, Koubaa M, Hammami F, Marrakchi C, Rekik K, Ben Jemaa T, et al., editors. Performance of an easy and simple new scoring model in predicting multidrug-resistant enterobacteriaceae in community-acquired urinary tract infections. *Open forum infectious diseases*; 2019: 6(4):ofz103. Oxford University Press US. doi: 10.1093/ofid/ofz103 30949542 PMC6441566

[41] Linhares I, Raposo T, Rodrigues A, Almeida A. Incidence and diversity of antimicrobial multidrug resistance profiles of uropathogenic bacteria. *BioMed research international*. 2015;2015:354084. doi: 10.1155/2015/354084 25834814 PMC4365316

[42] Bischoff S, Walter T, Gerigk M, Ebert M, Vogelmann R. Empiric antibiotic therapy in urinary tract infection in patients with risk factors for antibiotic resistance in a German emergency department. *BMC infectious diseases*. 2018;18(1):1–7. doi: 10.1186/s12879-017-2892-9 29373965 PMC5787273

[43] Leski TA, Taitt CR, Bangura U, Stockelman MG, Ansumana R, Cooper WH, et al. High prevalence of multidrug resistant Enterobacteriaceae isolated from outpatient urine samples but not the hospital environment in Bo, Sierra Leone. *BMC infectious diseases*. 2016;16(1):1–9. 27090787 10.1186/s12879-016-1495-1 PMC4836052

[44] Milovanovic T, Dumic I, Veličkovic J, Lalosevic MS, Nikolic V, Palibrk I. Epidemiology and risk factors for multi-drug resistant hospital-acquired urinary tract infection in patients with liver cirrhosis: single center experience in Serbia. *BMC infectious diseases*. 2019;19(1):1–10. doi: 10.1186/s12879-018-3567-x 30755176 PMC6373165

[45] Hantalo AH, Taassaw KH, Bisetegen FS, Mulate YW. Isolation and Antibiotic Susceptibility Pattern of Bacterial Uropathogens and Associated Factors Among Adult People Living with HIV/AIDS Attending the HIV Center at Wolaita Sodo University Teaching Referral Hospital, South Ethiopia. *HIV/AIDS* (Auckland, NZ). 2020;12:799–808. 10.2147/HIV.S244619 PMC7708265 33273865

