# Supplementary material for: Antimicrobial resistance trend of bacterial uropathogens at the university of Gondar comprehensive specialized hospital, northwest Ethiopia: A 10 years retrospective study
Source: PLoS One. 2022 Apr 11;17(4):e0266878. doi: 10.1371/journal.pone.0266878 (PMC9000029; doi:10.1371/journal.pone.0266878)
Supplement: Attachment — Submitted filename: PLOSREVIEW.docx [file pone.0266878.s001.docx]

PLOS ONE RVIEW

LINE 60- urinary tract infection is classified into upper urinary tract and lower urinary tract infections. Pyelonephritis and kidney infection are almost same.

61.62- suprapubic tenderness is a sign .

62.63- could be corrected as bacterial causes as UTI can be due to fungal species like candida

68- adherence to urethral mucosa ? is it unique for female sex alone?

69- kindly clarify the usage of the term “directly associated”. Doesn’t sound right.

74- you mean Ethiopia alone?

78- suggest usage of appropriate empirical treatment rather than appropriate treatment alone as most UTIs are treated empirically based on prevalent antimicrobial pattern.

81.82.83-sentence can be reframed to include trend over 10 years between 2009 and 2019

86- study period should include the period from which the study sample is selected rather than the period at which the data is collected.

92- was it a manual retrieval or retrieval from computerized data?

93,94- did the study included patients of all ages from birth to the maximum age recorded? If so any difference in the criteria for interpreting the results was applied between pediatric UTI and adult UTI? Did the study included out patients or inpatients or both?

96- what was the total number of samples recorded in the log book and how many were excluded based on your exclusion criteria. how many were excluded because of lack of information like age and sex in spite of having positive culture reports, which can influence the analysis?

111- “organisms” would be better term rather than” colonies” with significant

125- which version of CLSI guidelines? As the guidelines get revised periodically , kindly be more specific.

127. “The quality and completeness of patients’ urine culture records, as well as antimicrobial susceptibility results, were checked”. Can you clarify the statement as it is repeated again in the next sentence.

135 UTI suspected patients? According to the exclusion criteria patients without antimicrobial susceptibility testing were excluded form the study. Then how come they are included in the results? Kindly clarify

152,153- there is no information about antibiotic susceptibility in table 2 while it has been cited here. Highly irrelevant.

156 table took looks very irrelavent what is the meaning of caculating the percentage with the over all urinary samples when it is already calculated for positive cultures alone?

185 table could have been better with the explanation for numbers and percentages in the heading row.( does it mean the percentage positive out of total numbers tested?)

192- kindly explain the methodology by which this trend is obtained in the methodology section itself. how this resistance percentage was calculated? And this is for all organisms? One should understand that CLSI guidelnies get changed every year and the antimicrobial susceptibility testing and reporting also changes. does this trend show the prevalence of MDR pathogens over years? No clear information . calculating over all prevalence for ten years doesn’t add much to the community. Not sure whether this might help in surveillance.

201. kindly remove the statement threat as we have more severe threats than UTI S.

211. there is a huge difference in prevalence from elderly in the previous study and younger age group in the current study. Did the earlier study include all patients from birth? Clarify? If so, one cannot compare

213,214,215 what is the relevance of these statements to the current study?

236 isolates exceeded 50 percent? Statement or poorly framed and unclear

244.245 seems to be a loose statement. Is there any reference for local usage of antibiotics statment?

267 again seems to be a loose statement? Any reference for usage of antibiotic sin hiv patients?

274 earlier it was said that age groups 20- 30 was more affected, this statement is in contradiction with that. Kindly clarify. If its elderly then define elderly and cite the appropriate analysis for that in results and discussion
