# Supplementary material for: Antimicrobial resistance trend of bacterial uropathogens at the university of Gondar comprehensive specialized hospital, northwest Ethiopia: A 10 years retrospective study
Source: PLoS One. 2022 Apr 11;17(4):e0266878. doi: 10.1371/journal.pone.0266878 (PMC9000029; doi:10.1371/journal.pone.0266878)
Supplement: Attachment — Submitted filename: Point by point response to reviewors.docx [file pone.0266878.s002.docx]

**Response to reviewer(s)**

A. To PLOS ONE RVIEW

We are pleased that you, the PLOS ONE editorial team, offered us an invitation to revise our manuscript for the PLOS ONE journal. Also, we appreciate you for assigning such talented and qualified reviewers to our manuscript. We believed that their valuable comments helped us to improve our work.

1. **Line 60- Pyelonephritis and kidney infection are almost same.**

**Response**: Yeah, these terms are referring to infections of the upper urinary tract. We have revised and corrected it.

2. **Line 61.62- suprapubic tenderness is a sign.**

**Response**: “Suprapubic tender ness” has been included in a list of signs in many articles including the cited article. But now we have amended as sign and symptoms.

3. **62.63- could be corrected as bacterial causes as UTI can be due to fungal species like candida**

**Response**: Thanks for your suggestion, and now we have amended it.

.

4. **68- Adherence to urethral mucosa? is it unique for female sex alone?**

**Response**: Thank you for raising this issue. It is not unique for female sex alone, therefore, we have amended the sentence

5. 69- Kindly clarify the usage of the term “directly associated”. Doesn’t sound right.

**Response**: Thanks! We have revised and amended. As UTIs are among the most frequently treated infections, especially among women, it is obvious that exposure or usage of antibiotics will be increased, which intern hastens the emergence of antibiotic resistance. Hence these infections and drug resistance can have direct association.

6. **74- You mean Ethiopia alone?**

**Response**: No, we don’t mean that Ethiopia is the only one affected by these factors. The mentioned problems are also reported in many countries. These practices have been widely perpetuated in Ethiopia because of loose control system and wide availability of drugs without prescription in Ethiopia. There is a minimal attention to prevention of antimicrobial resistance in the country and awareness about the misuse or/and overuse of antibiotics in the community.

7. **78- Suggest usage of appropriate empirical treatment rather than appropriate treatment alone as most UTIs are treated empirically based on prevalent antimicrobial pattern.**

**Response**: Thank you for your suggestion, and now we have amended it.

8. 81.82.83-sentence can be reframed to include trend over 10 years between 2009 and 2019. **Response**: Amended as suggested but it is from 2010 to 2019, not 2009 to 2019.
9. 86- study period should include the period from which the study sample is selected rather than the period at which the data is collected.

**Response**: The period is included as per the comment and the sentence is written as “A Hospital-based retrospective cross-sectional study was conducted by retrieving laboratory record of ten years (2010-2019) data from January 1^st^ to February 28^th^, 2020 at the UoGCSH, Gondar, Ethiopia”

10. 92- Was it a manual retrieval or retrieval from computerized data?

**Response**: We have retrieved the data manually from the Laboratory paper-based record book

11. 93, 94- did the study included patients of all ages from birth to the maximum age recorded? If so any difference in the criteria for interpreting the results was applied between pediatric UTI and adult UTI? Did the study include outpatients or inpatients or both?

**Response**: Yes, this study included UTI patients irrespective of age group as per the inclusion criteria (having a complete record of the variables mentioned in the inclusion criteria). There were not any different criteria for interpreting UTI between pediatric and adults or any other age group. The microbiology laboratory at the UoGCSH uses growth of 10^5^ CFU/ml as a criterion for defining significant bacteriuria. We have used a record-based data and not able to identify between outpatients and inpatients. We could not determine the number of outpatients and inpatients as the origin of specimen was not recorded in the laboratory result registration book. We know that the UoGCSH laboratory receives urine for culture from inpatient and outpatients, therefore, we believe that both inpatients and outpatients were included in this study.

12. 96- What was the total number of samples recorded in the log book and how many were excluded based on your exclusion criteria. how many were excluded because of lack of information like age and sex in spite of having positive culture reports, which can influence the analysis?

**Response**: We have collected and analyzed records, which have full data of our target variables but the total number of the recorded urine culture results were 4209, of which 4088(included for analysis in this study) and 121 (rejected from this study because of missing of one or more of our inclusion criteria). Herewith we have mentioned the number and reason for exclusion in a table below.

| Number of samples rejected N=121 | Reason for rejection |
| --- | --- |
| 7 | Age was not recorded or missed |
| 18 | Sex was not recorded or missed |
| 83 | Age and sex were not recorded or missed |
| 11 | Culture result was not recorded |
| 2 | Isolated organism was not recorded |

13. 111- “organisms” would be better term rather than” colonies” with significant

**Response**: Thank you for your suggestion. We have changed it to “organisms”.

14. 125- Which version of CLSI guidelines? As the guidelines get revised periodically, kindly be more specific.

**Response**: Yes, it is known that CLSI is revised in a year basis, and the laboratory uses yearly uprated versions of the CLSI. So, a single year CLSI guideline has not been used.

15. 127. “The quality and completeness of patients’ urine culture records, as well as antimicrobial susceptibility results, were checked”. Can you clarify the statement as it is repeated again in the next sentence?

**Response**: Thank you for pointing this out. We have corrected it.

16. 135 UTI suspected patients? According to the exclusion criteria patients without antimicrobial susceptibility testing were excluded from the study. Then how come they are included in the results? Kindly clarify.

**Response**: We used “UTI suspected patients” to express those patients with urinary tract complain and for whom urine culture test was requested.

Patients without antimicrobial susceptibility testing result were excluded from the study which mean patient with significant bacteriuria but no AST recorded. This means for patients with **significant bacteriuria**, although records of age, sex, and **significant bacteriuria** were available, they were excluded if records of their antimicrobial susceptibility test results were not available, if result was “significant bacteriuria”, AST must also be recorded to include into the study. This does not mean that the isolate must be tested to all the mentioned antimicrobial agents. The isolated organisms were tested to different agents depending on their availability in the laboratory. Records without significant bacteriuria were also included if all variables age, sex and culture result (no significant bacteriuria) were recorded.

17. 152,153- There is no information about antibiotic susceptibility in table 2 while it has been cited here. Highly irrelevant.

**Response**: Thank you! We have modified it.

For understanding we have mentioned that “only two *Morganella morganii* (*M. morganii*), both resistant to ampicillin and amoxicillin-clavulanate, and either of which being resistant to gentamycin, trimethoprim-sulfamethoxazole, tetracycline, ciprofloxacin, tobramycin, and nalidixic acid were recorded (Table 2)”

Here, the antimicrobial susceptibility test was not written because only 2 *Morganella morganii* isolates were isolated and tested and their susceptibility results for the tested antibiotics was as mentioned in the text. Their susceptibility was omitted from the table 4 to minimize crowding. The table 2 was cited for the species of isolated organisms, not to the antimicrobial susceptibility testing. But now we have canceled it to avoid confusion.

18. 156 table took looks very irrelevant what is the meaning of calculating the percentage with the overall urinary samples when it is already calculated for positive cultures alone?

**Response**: We have used Table -2 to indicate the proportion of each bacterial species from the total isolates. However, as it is suggested, the proportion from the total number of urine sample (column 3) could have been omitted and now corrected.

19. 185 table could have been better with the explanation for numbers and percentages in the heading row (does it mean the percentage positive out of total numbers tested?)

**Response**: Thank you for your suggestion. We have reported the resistant isolates using frequencies and percentages. Each percentage were calculated by dividing the number of the same bacteria (e.g., *E. coli*) reported as resistant to a particular antibiotic agent (e.g., Ceftriaxone) from the total number of that bacteria (resistant or sensitive) tested against the same agent.

20. 192- kindly explain the methodology by which this trend is obtained in the methodology section itself. how this resistance percentage was calculated? And this is for all organisms? One should understand that CLSI guidelnies get changed every year and the antimicrobial susceptibility testing and reporting also changes. does this trend show the prevalence of MDR pathogens over years? No clear information. calculating over all prevalence for ten years doesn’t add much to the community. Not sure whether this might help in surveillance.

**Response**: Thank you for your questions. We have explained the trend in the method based on your suggestion. In fact, we have explained the overall resistance trend on a year basis. We have shown the plot of resistance pattern (it may be the sum of mono resistant and/or MDR isolates) reported over ten years but not the trend of MDR isolates.

21. 201. Kindly remove the statement threat as we have more severe threats than UTI S.

**Response:** corrected accordingly**.**

22. 211. there is a huge difference in prevalence from elderly in the previous study and younger age group in the current study. Did the earlier study include all patients from birth? Clarify? If so, one cannot compare.

**Response**: The studies we used here for comparison with our result have similar population to our study population.

23. 213,214,215 what is the relevance of these statements to the current study?

**Response**: These have been included to mention the possible rationale that support our result in which UTI was higher in the elderly populations.

24. 236 isolates exceeded 50 percent? Statement or poorly framed and unclear

**Response**: The statement has been rewritten to make it clear.

25. 244.245 seems to be a loose statement. Is there any reference for local usage of antibiotics statment?

**Response**: We have used the term “local usage” to refer the national guidelines for antimicrobial use (we have changed the term to national guidelines for antimicrobial use. Different countries use their own guidelines for antimicrobial use as per their countries’ situations like economic, or distribution of antimicrobial resistance. (Food E. Medicine and Healthcare Administration and Control Authority. Continuing Professional Development (CPD) Guideline for Health Professionals in Ethiopia Addis Ababa: FMoH. 2013. https://www.who.int/selection_medicines/country_lists/Ethiopia_STG_Hosp.pdf)

26. 267 again seems to be a loose statement? Any reference for usage of antibiotics in hiv patients?

**Response**: It is known that HIV patients are at greater risk of other infections and hence, they are likely to get antibiotics for these infections. Such a high risk of infection and resulting antibiotic treatment may increase the risk of resistance. The WHO has recommended prophylactic prescription of trimethoprim-sulfamethoxazole for HIV infected children. The cited reference itself has described it.

Reference: ([https://doi.org/10.1371/ journal.pone.0243054](https://doi.org/10.1371/%20journal.pone.0243054))

27. 274 earlier it was said that age groups 20- 30 was more affected, this statement is in contradiction with that. Kindly clarify. If its elderly, then define elderly and cite the appropriate analysis for that in results and discussion.

**Response**: As it is described in the result and table-1, the highest proportion of UTI suspected patients were in the age group 21–30 years (27.1%). However, the rate of infection was highest in the age group 61-70 years (47.4%) followed by patients of the age group above 70 years (44.8%). So, the point we have written in line 274 or in the conclusion is not conflicting. We think you may have seen in the line number -212, “the age group 21-30 was the most affected” but that was not our result rather it was a result reported in Addis Ababa, another part of Ethiopia and used here for comparison.

B. RESPOSES TO REVIEWER(S)
1. Reviewer #1: dear authors
   There are several lacunae in the methodology part with respect to inclusion and exclusion criteriae. more than one loose statement has been made without citations. quite a lot of errors seen in the discussion section too. kindly provide the details of analysis made for making out the trends in antimicrobial resistance over years. kindly go through the comments and make the necessary revisions

**Response:** We appreciate you for taking time to thoroughly review this manuscript and offer us your constructive comments and suggestions which will strengthen the quality of our work. We have revised the manuscript and made corrections as necessary based on your comments. We have tried to find and cite appropriate references to every information stated. We also have invited a professional English editor and so, the language and grammatical issues have been curiously amended.

2. Reviewer #2:  Comments:

In method section, sub-section Study area (Line 92), it is stated “We have collected ten years (2010-2019) retrospective data from the microbiology laboratory logbook”. This should describe more detailed e.g., mention how does the author confirm the adequate data and represent for each year period? Moreover, the author should also mention the consistency of practical work in the laboratory for ten years, i.e., method of susceptibility testing, antimicrobial disk used. I think this is very important point as the strength of the manuscript.

**Response:** we appreciate your concern about the adequacy and representativeness of the data. We have collected patients’ records which were complete record of the variables mentioned in the inclusion criteria within the whole study period. Moreover, representativeness of the collected data should not be the concern here because we have collected all the available data processed each year and contain complete record of the required variables, we did not take a sample.

Regarding the consistency of the laboratory work: The Hospital is one of the biggest pioneering hospitals in the country, Ethiopia. The laboratory performs microbiology culture constantly throughout the year and had no interruption over the study period. We know the laboratory as we are working with the hospital and one of the authors, Mr. Michael Getie, is manager of the microbiology laboratory. The UOGCSH microbiology laboratory had been using disc diffusion method of AST has been used in the laboratory. The antimicrobial agents could have been used up for a brief period until purchased but as the national antimicrobial treatment guideline was not changed long term discontinuation of antimicrobials has not occurred and antimicrobial disk used have been are mentioned in the method section. So, commonly prescribed discs have been used consistently.

3. In method section, sub-section population (Line 94-95), it is stated “all symptomatic patients who visited the hospital from 2010-2019 and had recorded urine culture results were the study population”. Please describe how the author performs the screening of the patient! This is important information to be added in the method section. Moreover, description about elaboration data with microbiology aspect is needed, as appear in Line 129-130: “The data were collected by investigators with quality and completeness checks throughout the collection period, at the end of data collection, and after entry to SPSS for statistical analysis.” Also, need to define the definition for trend of AMR in this study!

**Response:** As this study is based on a retrospective data, we have retrieved the record for urine cultures and collected the data of patients whose age, sex, urine culture result, and antimicrobial susceptibility results (for those with significant bacteriuria) have fully recorded. So, we have not screened patients. We had been cross-checking and re-checking data on a daily basis for the readability, correct data transcription to the data collection checklist, complete filling of the intended variables

4. In method section, the author should mention how the percentage calculation for resistance trend among antibiotic tested. What is denominator of the calculation? This information is still missing from the method section.

**Response:** the percentage of resistance was tested by dividing the resistant isolates to the total number of tested isolates to each antibiotic. The percentage of resistance trend was calculated by dividing the sum of resistant isolates in a year to the sum of isolates tested to each agent in the same year. E.g., the sum of resistant isolates identified in 2019/ number of urinary pathogens isolated tested in 2019.

5. In the inclusion criterion, I think it seems like “negative” statement or opposite from inclusion, please clarify.

**Response: W**e have included the patients’ data if the following hade ben completely recorded

- Age, Sex
- Urine culture result (both significant bacteriuria and no significant bacteriuria)
- Isolated uropathogen antimicrobial susceptibility result (for the result with significant bacteriuria)

6. In Line 110 – 121: Does it show microbiology procedure? Please write more clearly and elaborate with whole process of the study.

**Response:** it shows a precise procedure of the urine culture conducted in the microbiology laboratory, where the data was collected. But we have not gone through these laboratory procedures rather we have collected the result done by performing all these procedures as per the standard laboratory operating procedure and the Clinical Laboratory Standards Institute (for selection of antimicrobials and reporting result as susceptible and resistant)

7. Result:
   In line 134, the author mentioned about “UTI suspected patient”; but in population section, it was described “all symptomatic patients” (Line 94-95), which means not suspected patient. Please clarify for this sentence! If the author started with suspected patient, then provide the number of patients being suspected and mentioned how many of this become symptomatic patient with culture-proven UTI.

**Response:** First sorry for the inconvenience, and in the study area, patients showing signs and symptoms are suspected or presumptive UTI patients and will be supposed to give urine for urine culture for microbiological confirmation. When we suspected patients, we mean patients having signs and symptoms of UTI and all suspected patients were symptomatic patients in this study. so, now we have corrected as “UTI suspected” patients.

8. In table 2: the percentage is confusing and need to defined properly in method section (see the method comment). There is number of 4441, which indicate the number of total specimens, and number of 1072, which indicate the isolate included based on study criteria. Please explain for this two-different information!

**Response:** The number 4441 is the total number of collected patients’ data (both with significant bacteriuria and with no significant bacteriuria), while the number 1072 represents the total number of patients with significant bacteriuria or simply confirmed UTI patients) but all 4441 patients were included in this study. The % in the 3^rd^ column of table 2 is the percentage of each species of isolate from the total number of patients’ while the 4^th^ column is the percentage of each species of isolate to the total number of patients with significant bacteriuria or total number or organisms isolated in this study.

9. Lot of mistypes for species name i.e *Prouteus, Klebsella*. Author need to check all the typing for species name. Also, consistency for using italic formatting.

**Response:** We have revised and corrected the misspelled organisms.

10. In table 3 and 4: what does the number in bracket indicate? Please provide clear calculation for this table. What is the denominator for the calculation?

In these tables, the denominators indicate the total number of species of isolates tested against to each antimicrobial agent and in the last column the denominator is the total number of all isolates tested to each antimicrobial agent. The denominator is not constant because number of organisms tested varies. The numerator is the percentage of isolates which were resistant from the total tested organisms to a specific antimicrobial tested in a row. The numbers in bracket of in table 3 were the total number of each species isolate and we have canceled the numbers as it is found in table 2 and to avoid repetition.

11. I think the figure 1 and 2 is main finding of this study and should be attached in the body of manuscript, instead as supplementary figure. the result should more address the trend of bacterial and antimicrobial from 10 years observation, instead of mentioned single speces with resistance profile.

The tables were included in the body of the manuscript, but figures were attached as separate file according to the journal manuscript submission guideline.

Discussion:
I don’t see the discussion related with trend as the main title of the manuscript. Please correlate the finding with the term of Trend as mentioned with title of this study.

Response: We have tried to discuss the trend as stated in the discussion part (line 253-259)
