# Supplementary material for: Antimicrobial resistance trend of bacterial uropathogens at the university of Gondar comprehensive specialized hospital, northwest Ethiopia: A 10 years retrospective study
Source: PLoS One. 2022 Apr 11;17(4):e0266878. doi: 10.1371/journal.pone.0266878 (PMC9000029; doi:10.1371/journal.pone.0266878)
Supplement: Attachment — Submitted filename: New Microsoft Word Document.docx [file pone.0266878.s003.docx]

Recommendations

Most of the errors which have been pointed out have been corrected

The calculation of trends for individual species could have been a better parameter rather than calculating for overall species. The trend pattern suggests reduction in the percentage of resistance isolates in 2019 as compared to 2010. This is in contradiction to what is seen world wide and even from the same area reported by other authors. Since the CLSI guidelines change the testing pattern of antibiotics over years it would be better if the authors can state the definition of resistant isolates, like to which antibiotics does it refer to. And is this definition kept same for all the years? In such a case this study shows that there has been no increase in the antimicrobial resistance over 10 years which is really surprising.
