# Supplementary material for: Antimicrobial resistance trend of bacterial uropathogens at the university of Gondar comprehensive specialized hospital, northwest Ethiopia: A 10 years retrospective study
Source: PLoS One. 2022 Apr 11;17(4):e0266878. doi: 10.1371/journal.pone.0266878 (PMC9000029; doi:10.1371/journal.pone.0266878)
Supplement: Attachment — Submitted filename: Response to Reviewers.docx [file pone.0266878.s004.docx]

**Point by point response to reviewer(S)**

**Academic Editor/Reviewer 1:**

The resistance percentage is calculated for overall isolates and the trend shows it has been stable over years and has reduced now. I recommend you to clarify the exact criteria used for defining resistance isolates and whether it is applicable for all the years of the study, which is unlikely as clsi guidelines change every year.

**Response:** Thank you for your concern.

The criteria, that has been used to interpret the antimicrobial resistance by the laboratory in which our study was done, is the CLSI guideline. In fact, the CLSI is updated on a yearly basis and the laboratory uses the newly updated versions of CLSI each year. So, the results were reported as susceptible or resistant to the antimicrobial agents used as per the CLSI’s criteria and still no other criteria have been used in the laboratory. Up on release of new versions the CLSI the laboratory gets new versions timely each year and reports as per the new guideline. We are confident that the results are reported following the CLSI guideline and will not deviate the CLSI. E.g., in 2010 antimicrobial susceptibility test (AST) results were interpreted and reported based on 2010’s CLSI AST reporting criteria and that of 2019 results were reported following 2019’s version of CLSI. The number of organisms tested, the number and class of antimicrobial agents used could have a role in the slightly reducing resistance trend. The antimicrobial stewardship program is currently being implemented in the university of Gondar comprehensive specialized hospital and it might also be bringing the intended outcomes in reducing the antimicrobial resistance.

**Reviewer #1**:

1. Most of the errors which have been pointed out have been corrected the calculation of trends for individual species could have been a better parameter rather than calculating for overall species. The trend pattern suggests reduction in the percentage of resistance isolates in 2019 as compared to 2010. This is in contradiction to what is seen worldwide and even from the same area reported by other authors. Since the CLSI guidelines change the testing pattern of antibiotics over years it would be better if the authors can state the definition of resistant isolates, like to which antibiotics does it refer to. And is this definition kept same for all the years? In such a case this study shows that there has been no increase in the antimicrobial resistance over 10 years which is really surprising.

KINDLY provide more clarification of possible

**Response:** first we want thank you for considering our previous response. We also acknowledge your attention and critical review to make our work scientifically valuable.

2. **The calculation of trends for individual species could have been a better parameter rather than calculating for overall species**

**Response**:

This study was a retrospective study done by collecting a ten years laboratory data. The laboratory the antimicrobial discs used for antimicrobial susceptibility test (AST) were not consistently used throughout 10 years due to supply interruptions of the discs. In the absence of full stock of antimicrobial discs, the laboratory uses only few discs which are available at hand. The other is the frequency of species isolated and tested shows huge difference from too few (e.g., *Serratia* species = 4, *Providencia* species =5) to hundreds (e.g., *E. coli* =404) which makes the trend by species difficult to compare and conclude. So, calculating the species wise trend by species will not be important for conclusion. That is why we were not interested in analysis of species wise trend of resistance, but we have mentioned this as limitation.

3. The trend pattern suggests reduction in the percentage of resistance isolates in 2019 as compared to 2010. This is in contradiction to what is seen worldwide and even from the same area reported by other authors.

**Response**:

Yes, the trend was inconsistent and it seems that the percentage of resistance reported in 2010 was a little higher than the percentage of resistance reported in 2019. Multiple factors could have contributed for this seemingly contradiction. To mention some, the number of organisms tested, the number and class of antimicrobial agents used could have a role in the slightly reducing resistance trend. The tendency to replace antimicrobial agents with more potent discs could have reduced or maintain the resistance rate at steady level. For instance, there were higher frequency of ampicillin, amoxicillin use in 2010, 2011 and reduced the frequency of using these agents in recent years. Conversely, nitrofurantoin, which is a potent antibiotic used in the treatment of urinary tract infection, has not been used until 2014 and become increasingly used in the recent years. Moreover, 90.53% of meropenem disks were used for AST in the last two years (2018-2019). Amikacin has been used since 2017 and kanamycin has been used in 2018& 2019, which indicates drugs used for AST are changing. So, this phenomenon could have reduced the pooled percentage of resistance. The antimicrobial stewardship program is currently being implemented in the university of Gondar comprehensive specialized hospital and it might also be bringing the intended outcomes in reducing the antimicrobial resistance.

4. Since the CLSI guidelines change the testing pattern of antibiotics over years it would be better if the **authors can state the definition of resistant isolates**, like to which antibiotics does it refer to. And is this definition kept same for all the years? In such a case this study shows that there has been no increase in the antimicrobial resistance over 10 years which is really surprising.

**Response**:

We have collected data which have already been processed and recorded by the laboratory. So, we have collected antimicrobial susceptibility test (AST) results which have been reported as susceptible or resistant in each year and the laboratory uses each year’s update of the CLSI for interpretation of AST results as susceptible or resistant. E.g., in 2010 antimicrobial susceptibility test (AST) results were interpreted and reported based on 2010’s CLSI AST reporting criteria and that of 2019 results were reported following 2019’s version of CLSI.

**Reviewer #2:**

1. Thank you for addressing all the question adequate and properly.

2. However, table 3 and table 4 are still confusing to follow, especially number and percentage. In table 3, e.g Ampicillin, the total row number was 11 isolate/organism; 4 of 11 was Streptococcus spp. and 7 of 11 refer to Entercoccus spp. The proportion of resistance (percentage) for Streptococcus spp and Enterococcus spp, were 50%, 100%, respectively. In the author explanation was stated: "the percentage of resistance was tested by dividing the resistant isolates to the total number of tested isolates to each antibiotic." Referring to table 2, if the total number of isolates per organism, e.g Streptococcus spp was 26, the percentage calculation of 50% also did not fit. Kindly clarify! 3. My suggestion for point 2: if the number of isolate different according to the tested antibiotic, mentioned e.g: 4/8 (50%) or 7/7 (100%), to make the calculation clear; and put table note to highlight the meaning of the number.

**Response: Thank you dear reviewer.**

We have stated in our previous revision of the comments percentage of resistance was determined by dividing number of resistant isolates to the number of isolates. It is important to note that all isolates could have not been tested to each antibiotic. For instance, the total number of Streptococcus spp isolated in this study were 26, among these isolates only were 8 were tested against ampicillin and 50% of which were resistant to ampicillin. Why all isolated organisms were not tested could be due to availability of antibiotic discs, probably due to stock out or selection of priority agents.

We intended to minimize space if it is confusing, to avoid such a confusion we have corrected tables as per your comment.
