# Supplementary material for: Antimicrobial resistance trend of bacterial uropathogens at the university of Gondar comprehensive specialized hospital, northwest Ethiopia: A 10 years retrospective study
Source: PLoS One. 2022 Apr 11;17(4):e0266878. doi: 10.1371/journal.pone.0266878 (PMC9000029; doi:10.1371/journal.pone.0266878)
Supplement: Attachment — Submitted filename: comments on PONE-D-21-22966R2.docx [file pone.0266878.s005.docx]

Manuscript title: Antimicrobial resistance trend of bacterial uropathogens at the university of Gondar comprehensive specialized hospital, northwest Ethiopia: a 10 years retrospective study

Manuscript number PONE-D-21-22966R2

The authors tried to address most comments or concerns raised by the previous reviewers; however, still the authors should address the following comments to improve the quality of the manuscript:

Methods

The authors mentioned that the culture media and biochemical tests used. Since the laboratory logbook has been documented only patients’ age, sex, address, urine culture results and antimicrobial susceptibility test (AST) results and over the years the hospital may use different culture media and biochemical tests, so how did you trace the type of the media and biochemical tests used?

The authors are advised to perform PCR-based detection of the antimicrobial resistance genes in multidrug-resistant strains if applicable. (On the other hand, address this point to the study limitations).

Population: I think the study populations are not the patients that visited the hospital over the 10-year period rather it could be the urine culture results records. Please kindly explain about it.

Results:

One of the main of the study was to show the drug resistance patterns of the isolates over the specified time. However, the study lacks to reveal antibiotics/ class of antibiotics have a trend of increasing resistance over 10 years period. So, kindly request the authors to show the trend of resistance for each class of antibiotics on a yearly basis.

Still the calculation of trend over the years is not clear. It could be a better parameter and more explanatory if the calculation is based on the isolated species and a yearly base rather than considering the overall isolates.

**Antimicrobial resistance (AMR)**

It could be better the study showed that MDR, XDR and PDR isolates from each class of the antimicrobials and please illustrate each classes of antibiotics used and resistance patterns.

In figure 2, it showed the overall resistance species; it could be better if it depicts the resistance species and its trend over the years.

There are some calculations errors. For example table three, the first row the denominators should be 15 not 13. Please curiously address such issues.

In this table, I recommend to make one last row, label total isolates, and work out the total resistance isolates for each species. For example for CoNS, total tested isolates 172, resistance isolates 109 and rate of resistant was 63.37%.

The same is true for table 4. This table somewhat congested and please make it clear.

When the authors say LFGNR and NLFGNR, what it means? The authors mention LFGNR including E.coli, Klebsiella species, and Citrobacter and Enterobacter species. So what it stands? The same is true for NLFGNR. Unless you grouped together or indicated other that the stated species please avoid such jargon terminologies.

Discussions:

The authors are advised to illustrate the real impact of their findings in the managements of UTI in hospital as well as in the global community at large without repetition of results.

Minor issues:

LINE 51: More than 70% of isolates were resistant to…. Which isolates GPB or GNB?

LINE 69: 9) What is it number?

LINE88: ….retrospective cross-sectional….What kind of study design?

LINE 113-121: Where did the authors find the information/data? Did the data collected at the time of the tested performed? Please kindly explain where the source is.
