# Supplementary material for: Antimicrobial resistance trend of bacterial uropathogens at the university of Gondar comprehensive specialized hospital, northwest Ethiopia: A 10 years retrospective study
Source: PLoS One. 2022 Apr 11;17(4):e0266878. doi: 10.1371/journal.pone.0266878 (PMC9000029; doi:10.1371/journal.pone.0266878)
Supplement: Attachment — Submitted filename: Response to Reviewers.docx [file pone.0266878.s006.docx]

**Response to Reviewers**

First we would like to acknowledge the reviewers for the valuable comments and questions so that our manuscript will be mature scientific.

**Response to Reviewer-2**

Reviewer #2: Thank you for addressing the question properly. The last comment for reviewer2 has been answered properly, however refer to other reviewer comment to the different trend pattern, **my suggestion is author can explain the outbreak occurrence and/or surveillance method approach which may be useful as important point for discussion.**

**Response:** Thank you for considering our previous responses and your current suggestion! But this study was done to determine the burden of urinary tract infection (UTI) over a long (ten years) period retrospectively but outbreak was not seen nor surveillance of that outbreak were used to investigate occurrence of the disease. So, we have determined the prevalence of infection and resistance pattern of isolates retrospectively.

**Response to Reviewer-3**

We must acknowledge you for your comments and suggestions to improve the quality of our manuscript.

**Methods**

1. The authors mentioned that the culture media and biochemical tests used. Since the laboratory logbook has been documented only patients’ age, sex, address, urine culture results and antimicrobial susceptibility test (AST) results and over the years the hospital may use different culture media and biochemical tests, so how did you trace the type of the media and biochemical tests used?

**Response:** **Thank you for the concern:** The laboratory has standard operating procedure (SPO) in which the culture media used in the University of Gondar comprehensive specialized hospital laboratory and preparation of culture media is documented there and we have used that document as our source.

2. The authors are advised to perform PCR-based detection of the antimicrobial resistance genes in multidrug-resistant strains if applicable. (On the other hand, address this point to the study limitations).

**Response:** The study was retrospective and we directly took the record of the data. Even the laboratory has no molecular detection methods.

**We have accepted the comment and included in the limitation**

3. Population: I think the study populations are not the patients that visited the hospital over 10-year period rather it could be the urine culture results records. Please kindly explain about it.

**Response:** We have accepted your comment and corrected

**Results:**

4. One of the main of the study was to show the drug resistance patterns of the isolates over the specified time. However, the study lacks to reveal antibiotics/ class of antibiotics have a trend of increasing resistance over 10 years period. So, kindly request the authors to show the trend of resistance for each class of antibiotics on a yearly basis.

**Response: It is done in a class of antimicrobial agents on a yearly basis in table 5.**

5. Still the calculation of **trend over the years** is not clear. It could be a better parameter and more explanatory if the calculation is based on the isolated species and a yearly base rather than considering the overall isolates.

**Response:** the resistance trend in species was not done. Because, as it can be seen in table 5, antimicrobial agents were inconsistently used. E.g., carbapenem drugs were used only in the last two years. Isolates tested against penicillin (144) were not tested against aminoglycosides (100) and even among these isolates one could be tested to one but not to the other. This limited our analysis of trend on a species basis. Hence, it is more informative to analyze on a yearly basis and made a pooled resistance rate on a yearly basis can be conclusive.

**Antimicrobial resistance (AMR)**

6. It could be better the study showed that MDR, XDR and PDR isolates from each class of the antimicrobials and please illustrate each classes of antibiotics used and resistance patterns.

**Response:** Thank you!

To minimize congested nature of table, we have canceled the column of antimicrobial classes in other tables and mentioned classes of antimicrobial agents in **Table 5.**

As there are antimicrobial classes (such as Glycylcyclines, Monobactams…), that were not available in the laboratory and AST was not done to these agents, it is difficult to report XDR and PDR isolates. The definition is “MDR is defined as non-susceptibility to at least one agent in three or more antimicrobial categories. XDR is defined as non-susceptibility to at least one agent in all but two or fewer antimicrobial categories (i.e., bacterial isolates remain susceptible to only one or two categories). PDR is defined as non-susceptibility to all agents in all antimicrobial categories (i.e., no agents tested as susceptible for that organism)”. Hence, according to the definition we have only MDR isolates. The research is a retrospective study and we have analyzed and reported what had been done and recorded.

**(Reference**: Magiorakos AP, Srinivasan A, Carey RB, Carmeli Y, Falagas ME, Giske CG, Harbarth S, Hindler JF, Kahlmeter G, Olsson-Liljequist B, Paterson DL. Multidrug-resistant, extensively drug-resistant and pandrug-resistant bacteria: an international expert proposal for interim standard definitions for acquired resistance. Clinical microbiology and infection. 2012 Mar 1;18(3):268-81. <https://doi.org/10.1111/j.1469-0691.2011.03570.x>)

7. In figure 2, it showed the overall resistance species; it could be better if it depicts the resistance species and its trend over the years.

**Response:** The resistant species were mentioned in table -3 and table 4, but the trend of resistance to each species was not done because antimicrobial agents were not consistently used to test each isolates and over years. So, we showing the trend of resistance over the years was done. Seen in No 5.

8. There are some calculations errors. For example, table three, the first row the denominators should be 15 not 13. Please curiously address such issues.

**Response:** tables were revised and corrected as per the comment

9. In this table, I recommend to make one last row, label total isolates, and work out the total resistance isolates for each species. For example, for CONS, total tested isolates 172, resistance isolates 109 and rate of resistant was 63.37%

**Response**: It can be seen in the table different denominators indicating different number of isolates were tested against each antimicrobial agent. So, different antibiotics are difficult to add up. For example, only one isolate of CONs was tested against Cefoxitin while 17 were tested against Trimethoprim-sulfamethoxazole (SXT) and that one isolate could or could not be tested against SXT.

10. The same is true for table 4. This table somewhat congested and please make it clear.

**Response**: we understand that the table is congested because of the number of drugs used and species of isolates were diverse. It could be good to cancel the denominators, it was submitted that way in our 1^st^ submission and changed to the current form with comment.

11. When the authors say LFGNR and NLFGNR, what it means? The authors mention LFGNR including *E. coli*, Klebsiella species, and Citrobacter and Enterobacter species. So, what it stands? The same is true for NLFGNR. Unless you grouped together or indicated other that the stated species, please avoid such jargon terminologies.

**Response**: LFGNR-Lactose fermenting Gram negative rods, NLFGNR- None lactose fermenting Gram negative rods.

There were records in which the genus and species isolated was not specified and were reported as LFGNR and NLFGNR. This is a retrospective study done by copying the record from the laboratory. Hence it is difficult to add to the specified species and we put these isolates in separate columns.

Discussions:

12. The authors are advised to illustrate the real impact of their findings in the managements of UTI in hospital as well as in the global community at large without repetition of results.

**Response:**

This study figures out the burden of UTI and the rate of antimicrobial resistance to the available antimicrobials. So, the hospitals in the locality can understand which antibiotics can be used in imperial treatment and which agent should not be prescribed. The global community can also get the figure in the region.

The expected impact of the study was stated in the introduction part line 77-81, line 285-293 as well as in the conclusion (line 303-307).

Minor issues:

LINE 51: More than 70% of isolates were resistant to…. Which isolates GPB or GNB?

Response: Done as per the comment. It includes both gram positive and gram-negative isolates.

LINE 69: 9) What is it number?

**Response:** It was a typographical error and now corrected.

LINE88: …. retrospective cross-sectional…. What kind of study design?

**Response**: the study design is a retrospective study design and cross sectional was added to indicate the data collected period. it is corrected as retrospective study.

LINE 113-121: Where did the authors find the information/data? Did the data collected at the time of the tested performed? Please kindly explain where the source is.

**Response**: The data were collected from the University of Gondar comprehensive specialized hospital microbiology laboratory record book.
