# Supplementary material for: Antimicrobial resistance trend of bacterial uropathogens at the university of Gondar comprehensive specialized hospital, northwest Ethiopia: A 10 years retrospective study
Source: PLoS One. 2022 Apr 11;17(4):e0266878. doi: 10.1371/journal.pone.0266878 (PMC9000029; doi:10.1371/journal.pone.0266878)
Supplement: Attachment — Submitted filename: Review feedback.docx [file pone.0266878.s007.docx]

Manuscript Number PONE-D-21-22966 "Antimicrobial resistance trend of bacterial uropathogens at the University of Gondar comprehensive specialized hospital, northwest Ethiopia: a 10 years retrospective study"

Thank you for considering reviewing this work again.

Overall, the author tried to address all questions and comments.

Thank you for addressing the question properly.

I have no more reservations or comments. Please move forward.

Thank you
